# Draft Genome Sequence of *Cupriavidus* sp. Strain SK-4, a di-*ortho*-Substituted Biphenyl-Utilizing Bacterium Isolated from Polychlorinated Biphenyl-Contaminated Sludge

**DOI:** 10.1128/genomeA.00474-14

**Published:** 2014-05-22

**Authors:** Claudia Vilo, Michael J. Benedik, Matthew Ilori, Qunfeng Dong

**Affiliations:** a Department of Biological Sciences, University of North Texas, Denton, Texas, USA; b Department of Computer Science and Engineering, University of North Texas, Denton, Texas, USA; c Department of Biology, Texas A&M University, College Station, Texas, USA; d Department of Microbiology, University of Lagos, Lagos, Nigeria

## Abstract

*Cupriavidus* sp. strain SK-4 is a bacterium capable of growing aerobically on monochlorobiphenyls and dichlorobiphenyls as the sole carbon sources for growth. Here, we report its draft genome sequence with the aim of facilitating an understanding of polychlorinated biphenyl biodegradation mechanisms.

## GENOME ANNOUNCEMENT

Polychlorinated biphenyls (PCB) can cause serious environmental contamination due to their recalcitrance, high toxicity, and bioaccumulation in soil and sediments (1, 2). The utilization of microorganisms that are capable of metabolizing PCB compounds might become an efficient and less aggressive alternative for disposing of PCB pollutants (3). However, bioremediation methods often fail because of the accumulation of *ortho*- and *para*-substituted PCB compounds (1). *Cupriavidus* sp. strain SK-4 was originally isolated from PCB-contaminated tertiary lagoon sludge and is capable of growing on *ortho*-substituted CB congeners as a sole carbon source (1, 4). Furthermore, it can grow on all monochlorobiphenyls and also on dichlorobiphenyls, such as 2,2′-dichlorobiphenyl and 2,4′-dichlorobiphenyl (1, 2).

We have sequenced the *Cupriavidus* sp. strain SK-4 genome via Eureka Genomics (Hercules, CA) by using the Illumina Genome Analyzer IIx from paired-end libraries, with an average insert size of 221 bp. The sequencing produced 8,919,674 reads with a total length of 454,903,374 bp. Based on the estimated genome size of SK-4, the sequencing achieved about 58-fold coverage of the genome. The sequencing reads have an average length of 51 bp with good quality scores, as evaluated by the FastQC program (version 0.10.0 [http://www.bioinformatics.babraham.ac.uk/projects/fastqc/]). Assembly of the reads was performed with the SOAPdenovo2 software (5), using a *k*-mer size of 31 bp and a minimum scaffold length of 500 bp. The final 247 scaffolds assembled were annotated by using the Rapid Annotations using Subsystems Technology (RAST) server version 4.0 (6).

The assembled draft genome is 7,710,843 bp long. Its G+C content is 66.3%, and the genome contains 7,140 protein-coding genes and 49 tRNA genes. The metabolic capabilities that are in dominant proportions include the metabolism of amino acids (14%), carbohydrates (14%), cofactors (8.5%), and aromatic compounds (6.5%). Of special importance are genes related to the metabolism of aromatic compounds since they may include genes related to PCB degradation, such as those encoding aromatic dioxygenases (7). The main degradation route observed in PCB-utilizing bacteria involves a group of enzymes encoded in the *bph* operon (7). From the SK-4 draft genome sequence, we confirmed the existence of the *bph* operon genes, which are the same as the genes previously reported in the SK-4 catabolic plasmid (4). The *bph* operon genes are almost identical in their sequence and organization to those in *Burkholderia xenovorans* LB400 (4), and they are positioned in the order *bphA1-bphA2-bphA3-bphA4*-*orfx5*-*bphB-bphC-bphK-bphH-bphJ-bphI-bphD*. Both SK-4 and LB400 are among the few known bacterial species that can utilize di-*ortho*-substituted chlorobiphenyl compounds for growth (1, 8). In addition, the homologs of some *bph* genes were found elsewhere in the genome, indicating possible duplications. For example, three homologs of the *bphC* gene (encoding 2,3-dihydroxybiphenyl dioxygenase) were found on different genomic loci. The SK-4 draft genome sequence may assist in the improvement of PCB bioremediation methods.

### Nucleotide sequence accession numbers.

The draft genome sequence of strain SK-4 has been deposited at DDBJ/EMBL/GenBank under the accession no. JFJW00000000. The version described in this paper is the first version, JFJW01000000.
